# CBX Family Members in Two Major Subtypes of Renal Cell Carcinoma: A Comparative Bioinformatic Analysis

**DOI:** 10.3390/diagnostics12102452

**Published:** 2022-10-11

**Authors:** Anna Maria Grimaldi, Ornella Affinito, Marco Salvatore, Monica Franzese

**Affiliations:** IRCCS SYNLAB SDN, Via Emanuele Gianturco 113, 80143 Naples, Italy

**Keywords:** CBX family, kidney cancer, bioinformatic analysis, expression profiles, prognosis

## Abstract

The biological function and clinical values of Chromobox (CBX) family proteins in renal cell carcinoma (RCC) are still poorly investigated. This study aimed to compare the expression profiles and clinical relevance of CBXs between the two most frequent subtypes of RCC, clear cell renal cell carcinomas (ccRCC) and papillary renal cell carcinomas (pRCC), and to investigate whether CBXs would play a more or less similar role in the pathogenesis and progression of these RCC subtypes. Considering these two RCC populations in the TCGA database, we built a bioinformatics framework by integrating a computational pipeline with several online tools. CBXs showed a similar trend in ccRCC and pRCC tissues but with some features specific for each subtype. Specifically, the relative expressions of CBX3 and CBX2 were, respectively, the highest and lowest among all CBXs in both RCC subtypes. These data also found confirmation in cellular validation. Except for CBX4 and CBX8, all others were deregulated in the ccRCC subtype. CBX1, CBX6, and CBX7 were also significantly associated with the tumor stage. Further, low expression levels of CBX1, CBX5, CBX6, CBX7, and high expression of CBX8 were associated with poor prognosis. Otherwise, in the pRCC subtype, CBX2, CBX3, CBX7, and CBX8 were deregulated, and CBX2, CBX6, and CBX7 were associated with the tumor stage. In addition, in pRCC patients, low expression levels of CBX2, CBX4, and CBX7 were associated with an unfavorable prognosis. Similarly, CBX3, CBX6, and CBX7 presented the highest alteration rate in both subtypes and were found to be functionally related to histone binding, nuclear chromosomes, and heterochromatin. Furthermore, CBX gene expression levels correlated with immune cell infiltration, suggesting that CBXs might reflect the immune status of RCC subtypes. Our results highlight similarities and differences of CBXs within the two major RCC subtypes, providing new insights for future eligible biomarkers or possible molecular therapeutic targets for these diseases.

## 1. Introduction

Renal cell carcinoma (RCC) is an insidious malignancy. It is currently the tenth most diagnosed tumor in women and the sixth in men, making up about 4% of all malignant tumors [1]. With a 76% 5-year relative survival rate, RCC is the deadliest urological cancer, although its survival rate has markedly improved since a low of 46.8% in 1977. The survival rate strictly depends on the stage at diagnosis, with a 5-year relative survival of 93% for stage I, localized disease, 72.5% for stage II/III regional disease, and only 12% for stage IV metastatic disease [2]. Although the diagnosis and management of RCC have improved remarkably [3], its incidence is expected to increase worldwide [4]. Therefore, the disease still warrants further investigation for better prevention and management. RCC is a heterogeneous disease comprising a collection of different subtypes, characterized by distinct genetic abnormalities and molecular signatures reflecting the differences in the cell type, biology, and underlying molecular mechanisms [5]. The most frequent renal tumor subtypes are clear cell renal cell carcinomas (ccRCC) and papillary renal cell carcinomas (pRCC), accounting for 70–80 % and 10–15 % of all the RCCs [2], respectively. Although pRCC (overall 5-year survival rate of 80–90%) has a better prognosis than ccRCC (overall 5-year survival rate of 55–60%) [6], the overall prognosis for both subtypes remains limited.

Currently, the detailed molecular mechanisms of these two RCC subtypes, in terms of occurrence and development, are not yet fully understood, although the application of proteomics and multi-omics approach to tissues, cultured cells, and biological fluids has provided significant advancement in RCC prognostic significance assessment [7,8,9,10,11]. 

RCC tumorigenesis is a complex process involving genetic and epigenetic alterations. However, only a few types of RCCs can be explained by genetic predisposition, and epigenetic alterations in RCC subtypes are still poorly understood [12]. Therefore, understanding the roles of epigenetic modifications and underlying mechanisms in RCC could improve its diagnosis and clinical management.

Chromobox family genes (CBXs) are epigenetic regulators, and they are associated with a series of chromatin modifications that inhibit the transcription of target genes as crucial elements of Polycomb repressive complex 1 (PRC1) [13]. Eight members of CBX proteins have been identified in the human genome (CBX1–8), each of which possesses a single N-terminal chromodomain [14]. Based on their molecular structure, CBXs are divided into two subgroups: the HP1 group, which includes CBX1, CBX3, and CBX5; and the Polycomb group (PcG), with CBX2, CBX4, CBX6, CBX7, and CBX8, all characterized by C-terminal polycomb repressor box. These eight transcriptional repressors in the human genome control cell identity and development [15]. They ensure that their target genes are appropriately repressed during differentiation in a cell-type-specific manner, playing a vital role in developing and remembering specific cell states. Therefore, they are involved in regulating heterochromatin, gene expression, cell senescence, and developmental programs [16]. In particular, they play a pivotal role in maintaining stemness in embryonic stem cells and cell fate decisions [17]. Misregulation of CBX function—and, therefore, loss of cellular identity—can lead to developmental defects and cancer [18,19]. Thus, CBXs are described as epigenetic drivers in human cancers, and their deregulation has been significantly associated with cancer occurrence and progression [16,19,20]. However, there is no consistent pattern expression among different family members [21]. Moreover, the involvement of this family of epigenetic regulators in RCC remains poorly elucidated. For these reasons, we decided to apply bioinformatics analysis to stimulate and identify new research directions to investigate and compare the role of CBXs in tumorigenesis and the progression of two major RCC subtypes (ccRCC and pRCC).

## 2. Materials and Methods 

### 2.1. Samples Collection and Sequencing

Details about sample collection and sequencing procedures are reported in [10,22]. Briefly, all biospecimens were acquired by the Cancer Genome Atlas (TCGA) Resource Network. Surgically resected tumor specimens were collected from patients diagnosed with renal cell carcinoma (cRCC) and papillary renal cell carcinoma (pRCC) that had not received any prior treatment for their disease (chemotherapy or radiotherapy). Institutional review boards at each tissue source site reviewed the protocols and consent documentation and approved the submission of cases to TCGA. All patients signed informed consent. All tumors were staged according to the American Joint Committee on Cancer (AJCC). Pathology quality control was performed on each sample and subjected to independent pathology review to confirm that the tumor specimen was histologically consistent with the diagnosis and that the normal specimen contained no tumor cells. Tumor samples with ≥60% tumor nuclei and ≤20% or less necrosis were included in the study. The Tissue Source Sites (TSSs) contributing biospecimens are reported in [10,22].

RNA was extracted from tumor and normal tissue specimens using the DNA/RNA AllPrep kit (Qiagen, Hilden, Germany) and quantified by measuring Abs260 with a UV spectrophotometer. RNA was then analyzed via the RNA6000 nano assay (Agilent, California, USA) for the determination of an RNA Integrity Number (RIN), and only samples with RIN ≥7.0 were included in this study.

Libraries were prepared by using the Illumina mRNA TruSeq kit following the manufacturer’s directions and sequenced on the Illumina HiSeq 2000. mRNA sequence reads were aligned to the reference genome, and gene expression was quantified based on the gene models defined in the TCGA Gene Annotation File (GAF).

### 2.2. Power Analysis

A posteriori power analysis was performed to find out what power would be obtained for the specified effect size and sample size. Considering two groups (normal and tumor), we performed a two-tailed Wilcoxon–Mann–Whitney test with an effect size d = 0.5 and an alpha level of 0.05, obtaining a power of 0.97 for KIRC and 0.74 for KIRP. By considering more than two groups (4 tumor stages), we performed a one-way ANOVA with an effect size d = 0.25, an alpha level of 0.05, and a total sample size of 200, obtaining a power of 0.84.

### 2.3. GEPIA2

Gene Expression Profiling Interactive Analysis (http://gepia2.cancer-pku.cn/ (accessed on 20 December 2021)) is a user-friendly and interactive web resource for analyzing the RNA sequencing expression data of 9736 tumors and 8587 normal samples from the TCGA and the GTEx projects, using a standard processing pipeline [23]. We used GEPIA2 to analyze the tissue-wise expression of eight CBX family members in ccRCC and pRCC between tumor/normal tissues. The TCGA dataset was available within GEPIA2 (Kidney renal clear cell carcinoma, KIRC: 523 and Kidney renal papillary cell carcinoma, KIRP: 286). We selected only TCGA normal for differential analysis and plotting. We also profiled the expression of CBX genes based on the patient pathological stage. Statistical significance for differential gene analysis was estimated by one-way ANOVA and setting *p* value < 0.05. We also performed a multiple-gene comparison and obtained an expression matrix plot of CBX genes in two RCC subtypes.

We also used GEPIA2 to perform survival analysis by Mantel–Cox test to compare the survival contribution, in terms of overall survival (OS) and disease-free survival (DFS), of CBX genes in RCC subtypes. The median expression threshold was considered for splitting the high-expression and low-expression cohorts and a *p* value cut-off of 0.05 to determine significance. For CBXs, which gave significant results, we also performed survival analysis based on the expression status of CBX genes and plotted a Kaplan–Meier curve.

### 2.4. TIMER2.0

Correlations between tumor-infiltrating immune cells (TIICs) and CBXs were assessed by the Tumor Immune Estimation Resource platform (TIMER2.0, http://timer.cistrome.org/ (accessed on 20 January 2022)), which provides dedicated functions for analysis and visualization of TIICs across multiple cancer types associated with their clinical impact [24,25]. We selected CBXs as genes of interest via the “Gene module” to generate a functional heatmap that reported the correlation between CBXs expression values, from KIRC and KIRP TCGA datasets, with the immune infiltration cells. TIICs included B cells, CD4^+^ T cells, CD8^+^ T cells, dendritic cells, macrophages, and neutrophils. “Purity Adjustment” based on the partial Spearman’s correlation was selected to perform this association analysis.

### 2.5. cBioPortal

The cBioPortal for Cancer Genomics Portal (https://www.cbioportal.org/ (accessed on 21 January 2022)) is a free tool for interactive exploration of multidimensional cancer genomics datasets. We used it to analyze the frequency of CBX gene alteration and co-expression of eight CBX family members. KIRC-TCGA PanCancer (in total 512 samples) and KIRP-TCGA PanCancer (in total 283 samples) data were selected for analysis. A z-score with the range ±2 between tumor and normal conditions was chosen for mRNA expression values. For the correlation analysis of all possible combinations for the CBXs family, Spearman’s correlation coefficient >0.3 and the *p* value < 0.05 were considered for significance.

### 2.6. Protein–Protein Interaction (PPI) Network and Functional Enrichment Analysis

The protein–protein interaction network was generated with the GeneMania Cytoscape plugin [26]. As CBX3/6/7 were found to be the genes with the highest alteration rates in both RCC subtypes, we decided to focus network analysis only on these genes. Physical and co-expression interactions were selected, and the enrichment analysis (FDR ≤ 0.05) based on Gene Ontology (GO) Biological Process was performed. Pathway analysis was performed by using Reactome (REAC) database implemented in g:Profiler [27]. The threshold of 0.05 was considered for the statistical significance after the Bonferroni correction.

### 2.7. Cell Lines Validation

To validate our results in cell lines, we downloaded expression data from the Cancer Cell Line Encyclopedia (CCLE) (https://depmap.org/portal/ (accessed on 22 August 2022)), an extensive collection of expression and genetic data for human cancer cell models [28]. The portal contains expression data from non-cancerous cell lines, clear cell renal carcinoma, and renal cell carcinoma. Each cell line is identified inside this data base with DepMap ID (for details see Table S1). Expression data are reported as mean ± standard deviation. 

## 3. Results

### 3.1. CBX Family mRNA Transcriptional Levels and Clinicopathological Parameters

**Figure 1:**
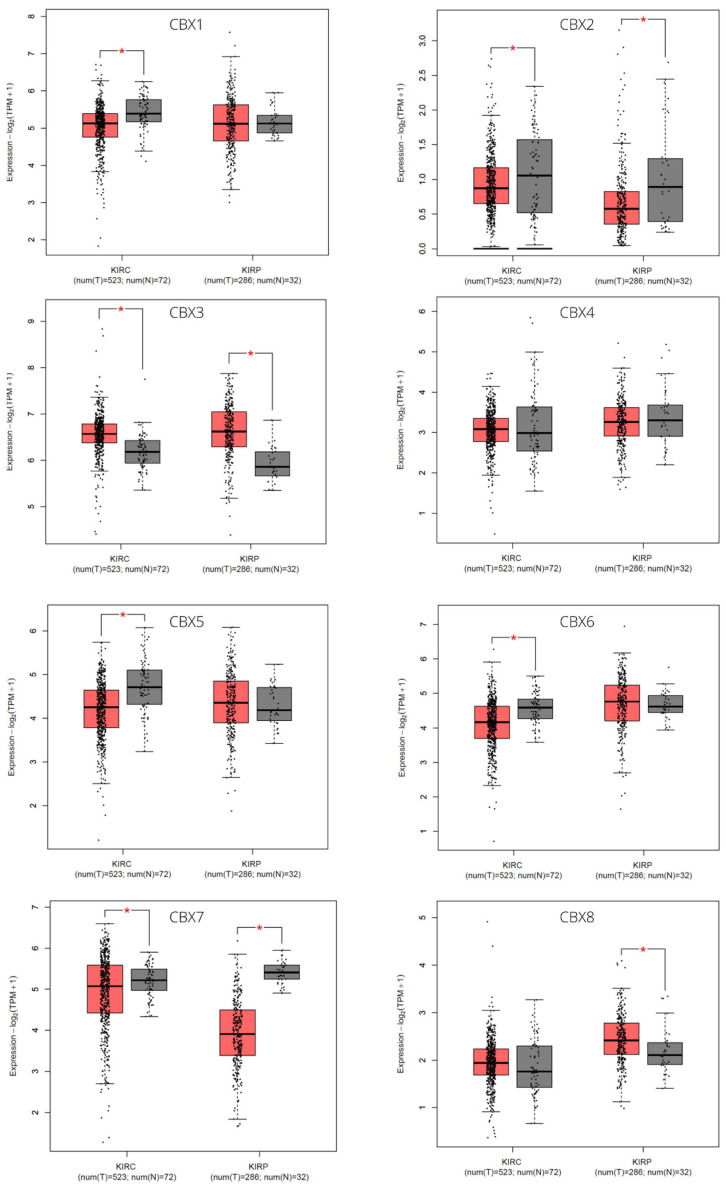
mRNA expression levels of CBX members in ccRCC (KIRC) and pRCC (KIRP) tissues and normal kidney tissues. *, *p* < 0.05. Analyses were performed using GEPIA2 * *p* < 0.05.

**Figure 2:**
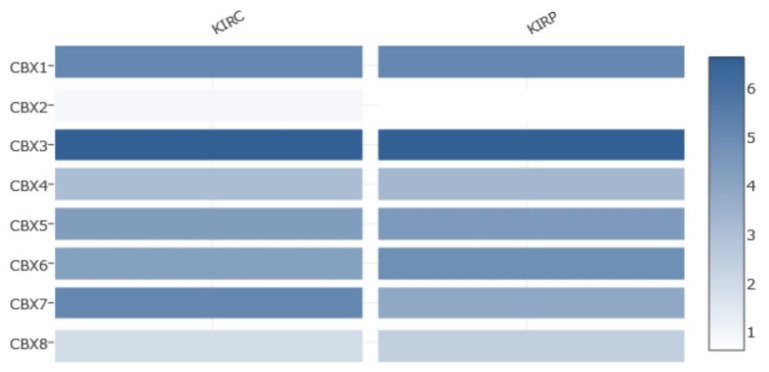
This feature provides expression matrix plots based on CBX genes. The density of color in each block represents the median expression value of a gene in a given tissue, normalized by the maximum median expression value across all blocks. Analyses were performed using GEPIA2 * *p* < 0.05. Abbreviations: KIRC: kidney renal clear cell carcinoma, KIRP: kidney renal papillary carcinoma.

**Figure 3:**
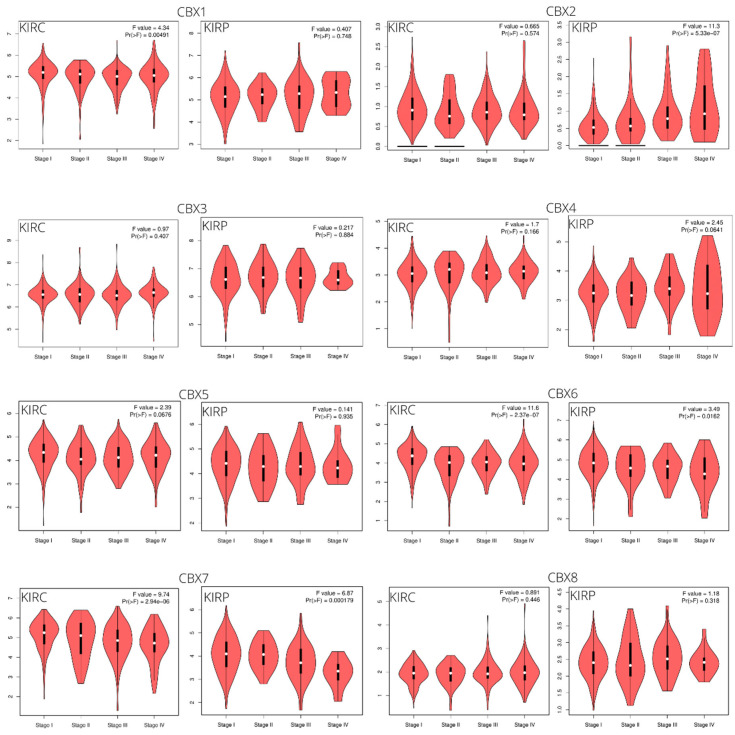
Association between CBX expression levels and clinical stage of ccRCC (KIRC) and pRCC (KIRP) patients. Analyses were performed using GEPIA2 * *p* < 0.05.

GEPIA2 was used to analyze the transcriptional levels of eight CBX genes in ccRCC and pRCC tissues compared with the normal kidney tissues. Clinical data of the selected populations, based on the TCGA database (KIRC and KIRP TCGA datasets), are summarized in Table 1. In ccRCC tissues compared with normal samples, the CBX1/2/5/6/7 were significantly downregulated in expression level, the mRNA expression of CBX3 was upregulated considerably, and no significant expression difference was found for CBX4/8 (Figure 1). In pRCC tissues, the CBX2/7 were significantly downregulated in expression level, the mRNA expression of CBX3/8 was significantly upregulated, and no significant expression difference was found for other CBX members (Figure 1). We also evaluated the relative expression levels of CBXs in comparing the two pathological conditions. We found that the CBXs relative expression followed a similar trend for both tumor subtypes: CBX3 was the highest among all CBXs, while CBX2 was the lowest (Figure 2). Additionally, the association of the expression of CBX genes with the tumor stage was analyzed. Specifically, the median expression of CBX2 showed an evident increase with more advanced tumor stages in pRCC, while the median expression of CBX7 showed decreasing trends in both RCC subtypes (Figure 3). Although without a net trend, the mRNA expression distribution of CBX1 in clear cell subtypes and CBX6 in both subtypes were significantly different among tumor stages (Figure 3).

### 3.2. Prognostic Value of CBXs Family mRNA Expression Levels in RCC Patients

**Figure 4:**
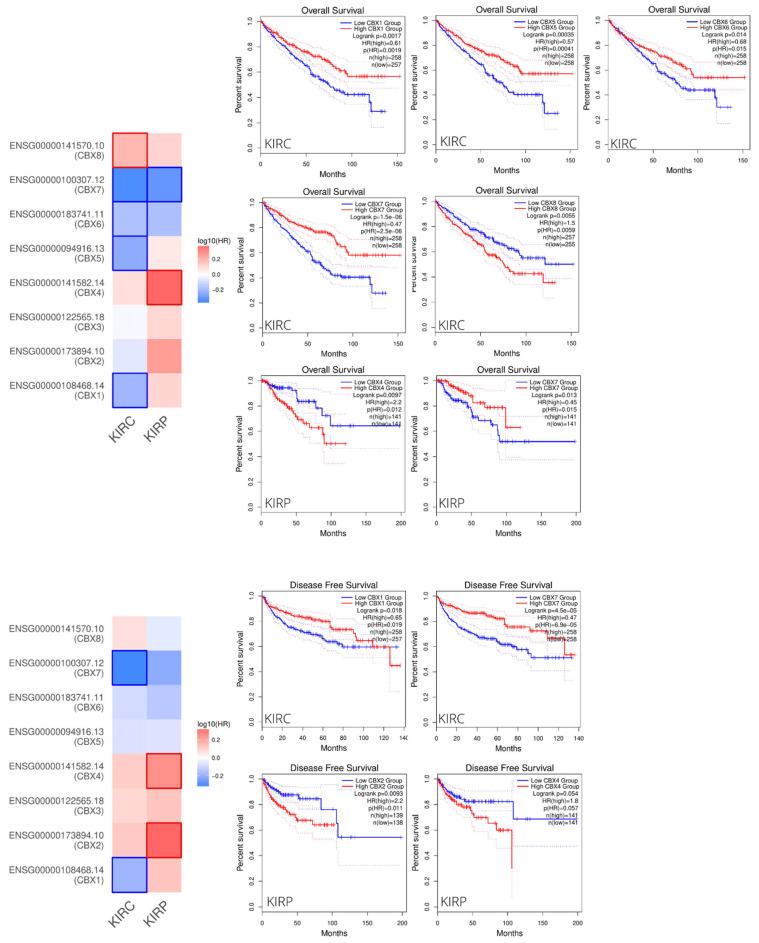
The prognostic impact of CBXs expression level is based on the survival heat map and Kaplan–Meyer curve. The heat maps show the hazard ratios in a logarithmic scale (log10) for different CBX genes. The red and blue blocks denote higher and lower risks, respectively. The rectangles with frames mean the significant unfavorable and favorable results in prognostic analyses (*p* < 0.05). The Kaplan–Meyer curves based on RCC subtypes have been reported only for CBXs with significant prognostic impact.

To screen for the prognostic impact of CBXs between RCC subtypes, the “Survival Map” module of GEPIA was used. In this way, we compared the survival contribution of CBX family members in ccRCC and pRCC in terms of OS and DFS. As shown in Figure 4, we denoted higher risk associated with CBX8 and lower risk with CBX1/5/6/7 in ccRCC. Similarly, CBX7 was associated with lower risk also in pRCC, while CBX2 and CBX4 were associated with a higher risk in the papillary subtype.

Specifically, the increased CBX8 and the decreased CBX1/5/6/7 expression values were significantly associated with shorter OS. Moreover, the low expression of CBX1 and CBX7 also had significantly unfavorable effects on DFS. Otherwise, in the pRCC group, the increased CBX4 and the decreased CBX7 expression levels were significantly associated with shorter OS. Furthermore, the high expression of CBX2 and CBX4 had significantly unfavorable effects on DFS.

### 3.3. Genetic Alteration and Co-Expression of CBX Family in RCC

**Figure 5:**
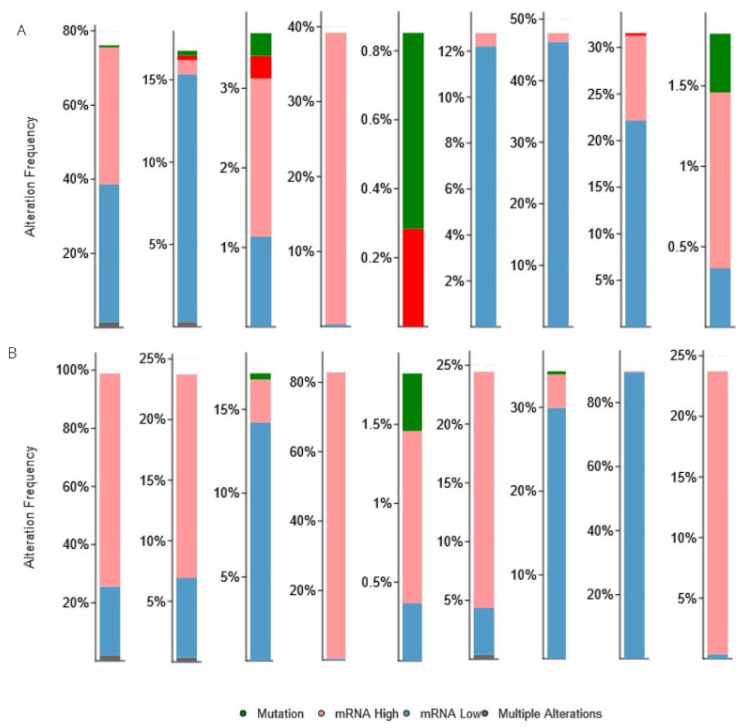
The alteration frequency and mechanisms for CBX family members in (**A**) ccRCC and (**B**) pRCC.

Using cBioPortal, we analyzed genetic alterations of the eight CBX proteins and their correlations with each other in ccRCC and pRCC populations. Results showed that the CBXs were altered in 76% of patients affected by ccRCC and in 99% of patients affected by pRCC (Figure 5A,B, respectively). Among eight CBXs, CBX3/6/7 were most altered in both RCC subtypes. Moreover, in both subtypes, the primary alteration type for CBX3 regarded high mRNA expression, while for CBX6/7 low mRNA expression.

**Figure 6:**
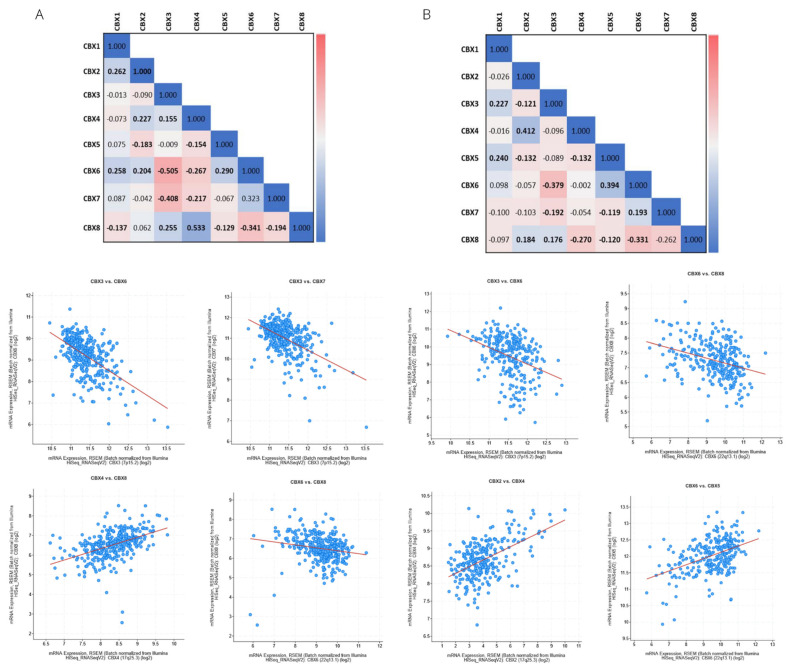
Correlations for mRNA expression of pairwise combinations of CBX family members in ccRCC (**A**) and pRCC (**B**) subtypes. Tables include Spearman’s Correlation coefficient (the bold font denotes *p* value < 0.05). The color scale interprets the correlation coefficient value. Plots show significant correlations (Spearman’s correlation coefficient >0.3 and *p* < 0.05).

Correlation analysis of the CBXs with each other revealed significant negative correlations between CBX6 with CBX3 (ccRCC: ρ = −0.51, *p* = 3.36^−24^; pRCC: ρ = −0.38, *p* = 8.78^−11^) and CBX8 (ccRCC: −0.341, *p* = 4.60^−11^; pRCC: ρ = −0.33, *p* = 2.05^−8^), for both RCC subtypes (Figure 6). Further, in the ccRCC group, we detected a positive correlation between CBX4 and CBX8 (ρ = 0.53, *p* = 3.20^−27^), and a negative correlation between CBX3 and CBX7 (ρ = −0.41, *p* = 1.43^−15^). Differently, in the pRCC group, positive correlations were found between the pairs CBX2 with CBX4 (ρ = 0.41, *p* = 1.17^−12^) and CBX5 with CBX6 (ρ = −0.39, *p* = 1.28^−11^).

### 3.4. Immune Cell Infiltration of CBXs in RCC Patients

To explore whether the expression of CBX genes was associated with the immune infiltration of RCC, we used the TIMER2.0 database (Table 2). The expression of CBX1 showed a significant positive correlation with the abundance of CD4^+^ T cells (ρ = 0.255, *p* =2.69^−8^), neutrophils (ρ = 0.295, *p* = 1.01^−10^) and macrophages (ρ= 0.344, *p* = 2.8^−14^) and a negative correlation with B cells (ρ = −0.0125, *p* = 7.23^−3^) in the clear cell population. Differently, it correlated with CD8^+^ T cells (ρ = 0.152, *p*= 1.43^−2^), neutrophils (ρ = 0.163, *p* = 5.52^−3^) and dendritic cells (ρ = 0.208, *p* = 7.75^−4^) in the papillary population. Regarding CBX2, it positively correlated with CD8^+^ T cells (ρ = 0.112, *p* = 1.62^−2^) and neutrophils (ρ = 0.213, *p* = 3.86^−6^) and negatively with B cells (ρ = −0.210, *p* = 5.64^−6^) in the ccRCC group. Otherwise, it was positively associated with B cells (ρ = 0.241, *p* = 9.13^−5^), CD8^+^ T cells (ρ = 0.236, *p* = 1.3^−4^), neutrophils (ρ = 0.198, *p* = 1.38^−3^) and dendritic cells (ρ = 0.184, *p* = 2.95^−3^) in the pRCC group. In ccRCC patients, the expression of CBX3 was positively associated with the abundance of neutrophils (ρ = 0.328, *p* = 5.34^−13^) and dendritic cells (ρ = 0.112, *p* = 1.59^−2^) and negatively associated with B cells (ρ = −0.149, *p* = 1.37^−3^), whereas in pRCC patients, its expression was related only to the abundance of neutrophils (ρ = 0.156, *p* = 1.21^−2^). The expression of CBX4 was positively associated with all of the assessed immune cells in both RCC subtypes (in details, for ccRCC: CD4^+^ T cells (ρ = 0.252, *p* = 3.98^−8^), neutrophils (ρ = 0.288, *p* = 2.79^−10^), dendritic cells (ρ = 0.243, *p* = 1.23^−7^); for pRCC: B cell (ρ = 0.211, *p* = 6.61^−4^), CD4^+^ T cells (ρ = 0.158, *p* = 1.11^−2^), CD8^+^ T cells (ρ = 0.256, *p* = 3.07^−5^), neutrophils (ρ = 0.327, *p* = 7.74^−8^) and dendritic cells (ρ = 0.328, *p* = 6.85^−8^). CBX5 mRNA was correlated with CD4^+^ T cells (ρ = 0.252, *p* = 4.02^−8^), macrophages (ρ = 0.387, *p* = 6.47^−18^), neutrophils (ρ = 0.364, *p* = 6.57^−16^) and dendritic cells (ρ = 0.220, *p* = 1.91^−6^) in ccRCC, while it was associated only with dendritic cells (ρ = 0.162, *p* = 9.13^−3^) in pRCC patients. The expression of CBX6 showed a positive correlation with CD4^+^ T cells (ρ = 0.318, *p* = 2.78^−12^), macrophages (ρ = 0.327, *p* = 6.30^−13^), neutrophils (ρ = 0.295, *p* = 1.01^−10^) and dendritic cells (ρ = 0.125, *p* = 7.33^−3^), and a negative correlation with B cells (ρ = −0.101, *p* = 3.09^−2^), while it was correlated with CD8^+^ T cells (ρ = 0.126, *p* = 4.35^−2^), macrophages (ρ = 0.188, *p* = 2.46^−3^), neutrophils (ρ = 0.145, *p* = 1.96^−2^) and dendritic cells (ρ = 0.220, *p* = 3.59^−4^) in pRCC patients. Except for dendritic cells and macrophages, CBX7 expression value was correlated with the abundance of the other immune cells (positively with B cells (ρ = 0.103, *p* = 2.78^−2^), CD4^+^ T cells (ρ = 0.192, *p* = 3.44^−5^), CD8^+^ T cells (ρ = 0.127, *p* = 6.20^−3^) and neutrophils (ρ = 0.181, *p* = 9.72^−5^) in the ccRCC population. Differently, it was positively correlated with all assessed immune cells in the pRCC population (B cells: ρ = 0.241, *p* =2.38^−2^; CD4+ cells: ρ = 0.226, *p* = 2.52^−4^; CD8+: ρ = 0.145, *p* = 1.98^−2^; neutrophils: ρ = 0.265, *p* = 1.66^−5^; macrophage: ρ = 0.168, *p* = 6.95^−3^; dendritic cells: ρ = 0.309, *p* = 4.12^−7^). Finally, CBX8 showed fewer associations with the abundance of immune cells: it was negatively related with CD8^+^ T cells (ρ = −0.116, *p* = 1.27^−2^) and positively with CD4^+^ T cells (ρ = 0.166, *p* = 3.37^−4^) in the clear cell subtype. In contrast, it was negatively related to dendritic cells (ρ = −0.134, *p* = 3.21^−2^) in the papillary subtype.

### 3.5. Protein–Protein Interaction (PPI) Network and Functional Enrichment Analysis

**Figure 7:**
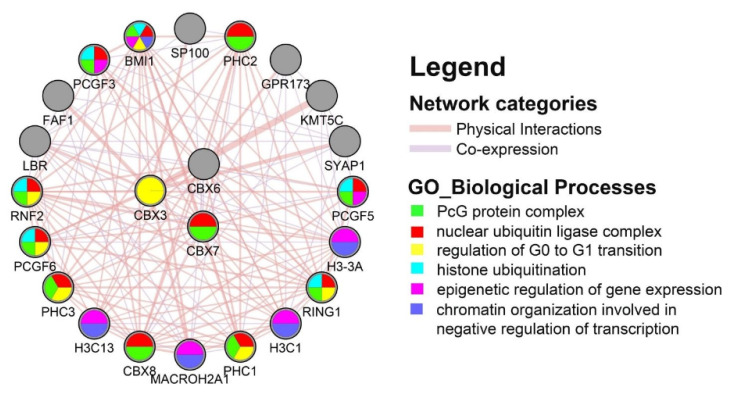
Protein–protein interaction network of CBX3, CBX6, and CBX7.

We decided to construct a functional protein-association network focusing on a subset of three CBX family members, specifically CBX3/6/7. For both assessed RCC subtypes, these three CBXs presented a similar profile: CBX3/6/7 showed the highest rates of genetic alteration, CBX3 mRNA level was upregulated, CBX6/7 associated with tumor stage, and CBX7 also associated with better prognosis. We first explored the top 20 genes linked to CBX members and then submitted the network for enrichment analysis. As shown in Figure 7, the PPI network was composed of 26 nodes and 138 edges. The top 20 functional partners were as follows: KMT5C, BMI1, RNF2, PHC2, PHC3, RING1, PCGF6, PCGF3, SYAP1, H3C13, PCGF5, LBR, H3-3A, FAF1, GPR173, PHC1, CBX8, H3C1, MACROH2A1, SP100. The GeneMANIA results also revealed that this network was most enriched for biological processes in protein ubiquitination, protein sumoylation, negative regulation of transcription, chromatin and nucleosome assembly, and G0 to G1 transition, while for cellular components, it was significantly enriched in PcG protein complex, chromatin, and nucleosome organization. From the Reactome analysis, we found that six pathways, including HSA-8939243 (RUNX1 interacts with co-factors whose precise effect on RUNX1 targets is not known), HSA-162582 (Signal Transduction), HSA-4655427 (Regulation of PTEN gene transcription), HSA-8878171 (Transcriptional regulation by RUNX1), HSA-427389 (ERCC6 and EHMT2 positively regulate rRNA expression), HSA-73772 (RNA Polymerase I Promoter Escape), were associated with CBX 3 and CBX6.

### 3.6. Cell Lines Validation

**Figure 8:**
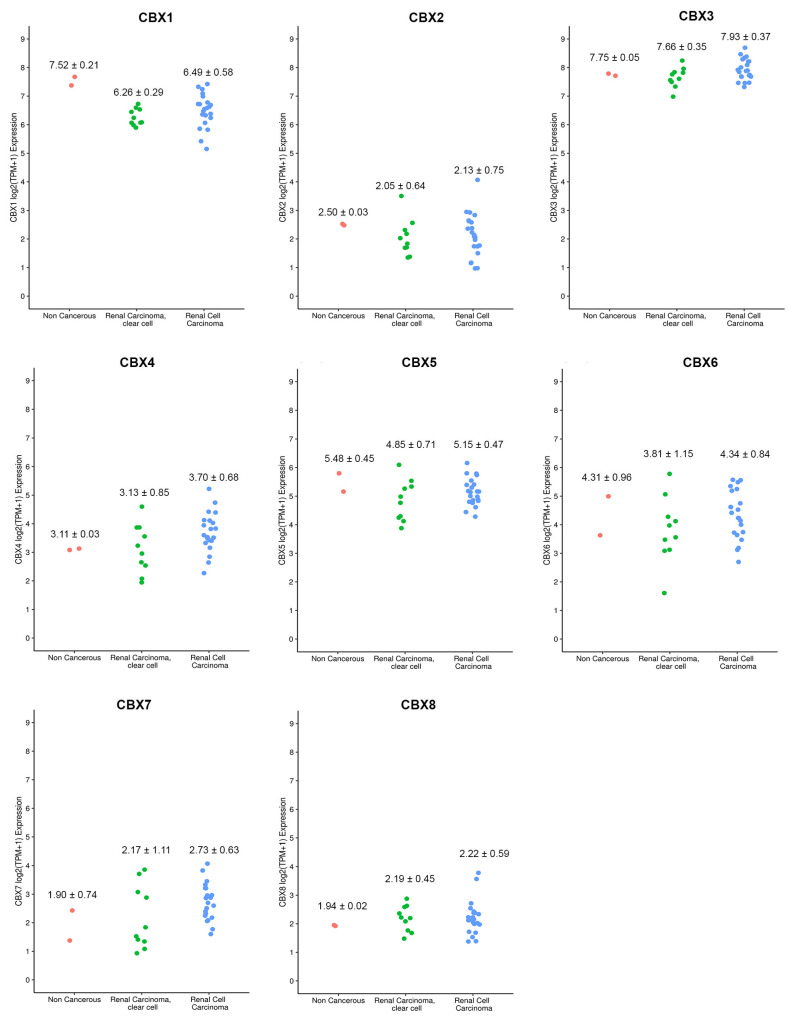
Distribution of gene expression values in non-cancerous, clear cell renal carcinoma, and renal cell carcinoma cell lines in different CBX genes. On the x-axis, the different kidney cell lines are reported; on the y-axis, the expression values for each cell line are shown. Each color identifies a specific cell type: pink for non-cancerous cell lines, green for clear cell line renal carcinoma, and blue for renal cell carcinoma. Each dot identifies a cell line (see Table S1). For each cell type, mean and standard deviation are also reported.

To validate our results, we used public kidney cell line data in CCLE. We evaluated the expression data from two non-cancerous cell lines, ten clear cell renal carcinoma and twenty-two renal cell carcinoma (Table S1). We observed that the expression trend of the tissues was also confirmed in cell lines (Figure 8). Specifically, we found that CBXs expression followed a similar trend for both tumor cell types (clear cell renal carcinoma and renal cell carcinoma). We also confirmed that CBX3 showed the highest expression values among all CBXs. In contrast, CBX2 showed the lowest ones (Figure 8).

## 4. Discussion

The critical role of epigenetic alterations in the development and progression of RCCs has been depicted [22,29,30]. However, epigenetic biomarkers for RCC are still not used in clinical practice, and targeted epigenetic therapies are under investigation [29]. As the critical components of the epigenetic regulatory complexes, CBX family proteins are involved in the development and progression of multiple cancers [18,31,32]. However, the distinct roles of the CBXs family in RCC have yet to be clarified. Recently, two papers have explored the clinical significance and biological function of CBXs in ccRCC. A first paper assessed the molecular mechanism of CBX4 on the development and progression of clear cell subtype in vitro and in vivo models [33], and a second used a bioinformatic approach to investigate the expression and prognostic value of CBXs [34]. To the best of our knowledge, no study has shown whether such similarities and differences exist in RCC subtypes. Based on the rapid development of second-generation sequencing technology and the establishment of a large number of databases, a comprehensive study of the CBXs family in RCC subtypes could help discover new biomarkers with diagnostic and prognostic potential or new therapeutic targets for this deadly disease. Therefore, in this work, we performed an integrated in silico approach using several bioinformatics tools to provide a comprehensive and comparative analysis of CBXs in the two most frequent RCC subtypes.

Overall, the relative expression levels of CBXs in ccRCC and pRCC subtypes showed a similar trend, with CBX3 and CBX2 having, respectively, the highest and lowest among all CBXs. However, the relevance and involvement of CBX members in the clinical evolution of the disease did not always show as much concordance in the two investigated tumor subtypes.

CBX1 has been reported to be upregulated in several cancers, such as breast [35], colorectal [36], and gastric cancer [37]. It has been detected to have higher expression in hepatocellular carcinoma (HCC), associated with worse clinical outcomes, and functions as an oncogene by interacting with the transcription factor HMGA2 to activate the Wnt/β-catenin signaling pathway in hepatocellular carcinoma [38]. Contrary to the pro-tumor effect assessed in HCC, here, we disclosed a potential antitumor effect of CBX1 in ccRCC, consistent with recent results reported by Zhu and colleagues [34]. Its significant deregulation was related to tumor stage and worse prognosis in ccRCC patients. In the papillary subtype, CBX1 did not seem to play a specific role in the disease because its expression profile appeared similar in tumoral and normal tissues and was not associated with progression or the prognosis of patients.

The overexpression of CBX2 has been reported in breast cancer, where it promoted the progression of the disease by the PI3K/AKT signal pathway [39]. Again, CBX2 has been found to regulate proliferation and apoptosis by phosphorylating YAS in hepatocellular cells [40]; it promoted the proliferation, invasion, and migration of gastric cancer by activating the YAP/beta-catenin pathway [41]. Our results highlighted CBX2 as the most down-expressed gene among all CBX family members in both RCC subtypes. Additionally, CBX2 provided prognostic information in the pRCC population, as it was associated with tumor stage and worse clinical outcomes.

Many studies have reported elevated expression of CBX3 in different human cancer tissues and proposed it as a predictor of unfavorable prognosis for malignancies [42,43,44]. High CBX3 expression promoted glioma proliferation by targeting CDKN1A [44]. Moreover, CBX3 binding activity to methylated histone H3K9 was required to promote the proliferation of colorectal cancer [45] and lung cancer [46]. Recently, some researchers found that high expression of CBX3 promoted ovarian cancer cell proliferation and was significantly related to unfavorable progression and chemoresistance, impacting the treatment outcomes of OV patients [47]. Our results showed similar biological behavior of CBX3 in both RCC subtypes. CBX3 emerged as the most overexpressed gene among all CBX family members in both RCC subtypes in ccRCC and pRCC. However, its overexpression did not correlate with cancer stages or patient prognosis for both subtypes.

CBX4 was overexpressed in breast cancer [48] and osteosarcoma [49]. Functionally, it promoted hepatocellular carcinoma via SUMOylating HIF-1α to upregulate vascular endothelial growth factor [50]. Silencing of CBX4 inhibited cell growth and metastasis in lung cancer by regulating the BMI-1 pathway [51]. More recently, the oncogenic activity of CBX4 has been demonstrated in ccRCC by inhibiting tumor suppressor KLF6 via interaction with HDAC1 [33]. CBX4 overexpression promoted tumor growth and metastasis, whereas CBX4 silencing resulted in the opposite phenotypes. In our study, CBX4 transcriptional level did not show significant changes between tumor and normal conditions in RCC patients. However, in the pRCC population, decreased CBX4 correlated with poor prognosis (shorter OS and DFS).

Regarding CBX5, its upregulation has been associated with increased cell proliferation and poor clinical prognosis [52,53]; furthermore, its downregulation was linked to a higher invasive potential of cancer cells in metastatic cells of colon cancer and thyroid carcinoma compared with non-metastatic cells [53]. Therefore, CBX5 deregulation can play dual mechanistic functions for cancer cell proliferation and metastasis suppression, and the underlying cellular mechanisms are not yet comprehensively known. Here, CBX5 mRNA was downregulated in ccRCC and was associated with a shorter survival time. In the papillary subtype, CBX5 transcriptional level was not significantly deregulated in tumor tissues and did not associate with other clinical parameters.

Our findings supported a different potential involvement of CBX6 in ccRCC and pRCC, and a similar tumor suppressive role for CBX7 in both assessed RCC subtypes. However, to date, the roles of these two CBX members appear unclear: depending on the tumor type or cellular context, they can act as oncogenes or tumor suppressors. For instance, significant downregulation of CBX6 was reported in breast cancer, where ectopic overexpression inhibited tumor cancer progression [54]. Otherwise, in liver cancer, high expression of CBX6 was associated with a worse prognosis [55]. In addition, regarding CBX7, experimental evidence provides contradictory results. CBX7 decreased expression has been reported for many carcinomas, such as breast [56], lung [57], colorectal [58], and thyroid [59]. In these malignancies, downregulation of CBX7 correlated with lower cancer aggressiveness and favorable prognosis, suggesting an oncosuppressor role of CBX7. Conversely, overexpression of CBX7 and its oncogenic effects have been reported in lymphoma [60], prostate [61], and ovarian cancers [62,63].

In addition, CBX8 has been hypothesized to have a contradictory role in various tumors. It was upregulated [64,65], associated with advanced tumor stages, and promoted cancer cell proliferation by inhibiting the p53 pathway in bladder cancer tissues [65]. Similarly, it promoted proliferation in colon cancer, but low expression was associated with poor prognosis of patients [66]. CBX8 has been reported to promote tumorigenesis in esophageal squamous cell carcinoma and to suppress metastasis by repressing Snail [67]. Here, CBX8 mRNA showed a significant increase between tumor and normal conditions in pRCC but not in ccRCC. However, only in this last subtype did CBX8 high expression have a worse prognosis.

Genetic alteration is a common phenomenon in various tumors, including RCC. All eight CBX family members were altered in RCC patients, and the total genetic alteration rate was 76% and 99% for ccRCC and pRCC, respectively. Several clues reveal the involvement of CBX methylation in tumors. For instance, CBX1/3/5 were found to act as methyl readers, which are involved in interpreting H3K9me3 signatures induced by H3K9 methyltransferases [17]. Furthermore, differentially expressed CBXs were found to have a strong association with SUMOylation of DNA methylation proteins in colorectal cancer [36]. In our analysis, CBX3/6/7 resulted in being the genes with the highest alteration rates in both RCC subtypes and their functions primarily related to the SUMOylation of DNA methylation proteins, chromatin organization proteins, RNA binding proteins, and signaling pathways that regulate pluripotency of stem cells, and transcriptional regulation by AKT, PTEN, and RUNX1 pathways. Of note, in agreement with these results, the involvement of the PTEN/Akt pathway in the tumor suppressive activity of CBX7 has been demonstrated in pancreatic cancer cells [68]. Furthermore, it has been reported that this protein regulates stem cell-like properties of gastric cancer cells via the activation of the AKT pathway [69].

RCC is considered an immunogenic tumor, and increasing findings support that immune cell infiltration is closely associated with clinical outcomes in RCC [70,71,72]. In this context, epigenetic regulation of innate immune response is an emerging field. CBX7 knockdown led to apoptosis of CD4^+^ T cells by hyperdemethylation of the FasL gene promoter and increased expression of FasL [73]. Moreover, experimental evidence has identified Cbx2 as a positive mediator in antiviral immunity, demonstrating its mechanistic involvement as an epigenetic modifier in the innate immune response. Precisely, it has been elucidated that CBX2 can bind to and recruit Jmjd3 to the Ifnb promoter in macrophages, leading to demethylation of H3K27me3 and increasing transcription of IFN-β, an essential factor involved in the innate antiviral immunity [74].

In our study, the expression of CBXs for both RCC subtypes correlated with the infiltration of different immune cells. Particularly, CBXs were associated mainly with neutrophils, CD4^+^ T cells, and B cells in ccRCC and with dendritic cells, neutrophils, and CD8^+^ T cells in the papillary subtype. Hence, we hypothesized that CBXs might regulate RCC tumor immunity through multiple immune cell populations. Therefore, in the future, it could be interesting to underpin the role of misregulated CBX1/4/5/7 in ccRCC and CBX4/7 in pRCC in affecting the immune status of RCC. We did not investigate the relationship between CBX members and mast cells. Mast cells are a kind of immune cell that can secrete diverse, active compounds to participate in the immune response. However, recent findings suggested that biological processes such as mast cell activation and mast cell-mediated immunity were regulated by the CBX family members in ovarian cancer [47].

We are aware that our study has several shortcomings. Firstly, the clinicopathological information of the RCC patients was obtained from public databases; secondarily, the study is fully bioinformatics-based and lacks further experimental validation in vitro and in vivo. Furthermore, we lacked research on the detailed mechanisms of individual CBX members in RCC. However, the concordance of expression trends between tissues and cell lines that emerged from in silico validation encourages future functional studies aimed at a deeper understanding of the involvement of this protein family in kidney cancer. Further studies are required to explore the specific mechanism between each CBXs member and kidney cancer.

## 5. Conclusions

In conclusion, we analyzed the expression, prognostic values, gene alteration, immune infiltration, and functional protein-association networks of different CBX family members in the two most frequent subtypes of RCC. In ccRCC, as in pRCC, increased expression of CBX3 and lower expression of CBX2 were detected. In addition, decreased expression levels of CBX6/7 were associated with tumor stage for both RCC subtypes. Increased expression values of CBX8 and decreased CBX1/5/6/7 were significantly associated with worse OS in ccRCC patients. Otherwise, in pRCC patients, increased expression levels of CBX4 and decreased CBX7 were significantly associated with shorter OS. Furthermore, a high genetic alteration rate of the CBXs family was observed in RCC. Moreover, individual CBX members were associated with different degrees of immune cell infiltration. Overall, these findings highlighted the interesting value of the CBXs family in the initiation and progression of renal cancer. Our results may provide new cues to select prognostic biomarkers or molecular targets among CBX family members in two major subtypes of RCC.

## Figures and Tables

**Table 1:** Clinical Data of Included Patients from the TCGA dataset.

Clinical Features	Category	KIRC, *n* = 533	KIRP, *n* = 290
Age	Median (range)	61 (26–90)	61 (28–88)
Gender	Female	188	76
	Male	345	214
Tumor Stage	I	267	140
	II	57	21
	III	123	29
	IV	84	11
	Not Available	2	89
T stage	T1	273	193
	T2	69	33
	T3	180	60
	T4	11	2
	TX	0	2
	Not Available	0	0
M stage	M0	422	95
	M1	79	9
	MX	30	171
	Not Available	2	15
N stage	N0	240	50
	N1	16	24
	N2	0	4
	NX	277	211
	Not Available	0	1
Vital status	Dead	175	44
	Alive	358	246
Laterality	Bilateral	1	2
	Left	251	160
	Right	281	127
	Not Available	0	1

KIRC: kidney renal clear cell carcinoma, KIRP: kidney renal papillary carcinoma.

**Table 2:** Spearman’s correlation between CBXs expression and the abundance of immune cell infiltration (TIMER2.0) in ccRCC and pRCC.

	**CBX1**		**CBX2**		**CBX3**		**CBX4**		**CBX5**		**CBX6**		**CBX7**		**CBX8**	
	ccRCC *n* = 533	pRCC *n* = 290	ccRCC *n* = 533	pRCC *n* = 290	ccRCC *n* = 533	pRCC *n* = 290	ccRCC *n* = 533	pRCC *n* = 290	ccRCC *n* = 533	pRCC *n* = 290	ccRCC *n* = 533	pRCC *n* = 290	ccRCC *n* = 533	pRCC *n* = 290	ccRCC *n* = 533	pRCC *n* = 290
B cell	−0.013	0.023	−0.210	0.241	−0.149	−0.030	0.002	0.211	−0.040	0.066	−0.101	0.044	0.103	0.141	0.095	−0.032
CD4^+^	0.255	0.080	0.073	0.059	0.024	0.024	0.252	0.158	0.252	0.067	0.318	−0.010	0.192	0.226	0.166	0.083
CD8^+^	0.067	0.152	0.112	0.236	0.048	0.038	0.099	0.256	−0.090	0.061	−0.055	0.126	0.127	0.145	−0.116	−0.112
Macrophage	0.344	0.011	−0.069	−0.083	0.061	−0.014	0.022	0.091	0.387	0.095	0.327	0.188	0.048	0.168	−0.049	−0.105
Neutrophil	0.295	0.163	0.213	0.198	0.328	0.156	0.288	0.327	0.364	0.116	0.295	0.145	0.181	0.265	0.028	−0.037
Dendritic cell	0.069	0.208	0.053	0.184	0.112	0.063	0.243	0.328	0.220	0.162	0.125	0.220	0.042	0.309	0.055	−0.134

Legend: The color pink indicates a significant positive correlation (*p* < 0.05, ρ > 0), the color blue indicates a significant negative correlation (*p* < 0.05, ρ < 0), and the color grey indicates no significant correlation (*p* > 0.05).

## Data Availability

The datasets analyzed in this study can be found in the TCGA database (https://portal.gdc.cancer.gov/ accessed on 28 September 2022). The datasets analyzed in this study for cell lines can be found in the Cancer Cell Line Encyclopedia (CCLE) (https://depmap.org/portal/ (accessed on 22 August 2022)). Each cell line is identified inside this database with DepMap ID. Specifically, DepMap ID for non-cancerous cell lines: ACH-001310, ACH-000049; DepMap ID for clear cell renal carcinoma: ACH-000313, ACH-000684, ACH-000234, ACH-000649, ACH-000411, ACH-000433, ACH-000907, ACH-000709, ACH-000272, ACH-000513; DepMap ID for renal cell carcinoma: ACH-000317, ACH-001398, ACH-000428, ACH-000429, ACH-000600, ACH-000459, ACH-000262, ACH-000484, ACH-000300, ACH-000250, ACH-000375, ACH-001194, ACH-000016, ACH-000171, ACH-000189, ACH-000457, ACH-000159, ACH-000385, ACH-000495, ACH-000246, ACH-000555, ACH-000046.

## Supplementary Materials

The following supporting information can be downloaded at: https://www.mdpi.com/article/10.3390/diagnostics12102452/s1, Table S1: Expression values of CBX1, CBX2, CBX3, CBX4, CBX5, CBX6, CBX7, CBX8 genes in different cell lines of non-cancerous, clear cell renal carcinoma and renal cell carcinoma cells.
